# Supplementary material for: Tonically active GABAergic neurons in the dorsal periaqueductal gray control instinctive escape in mice
Source: Curr Biol. Author manuscript; Available in PMC 2025 Jul 29. (PMC7617961; doi:10.1016/j.cub.2024.05.068)
Supplement: SI [file EMS206915-supplement-SI.pdf]

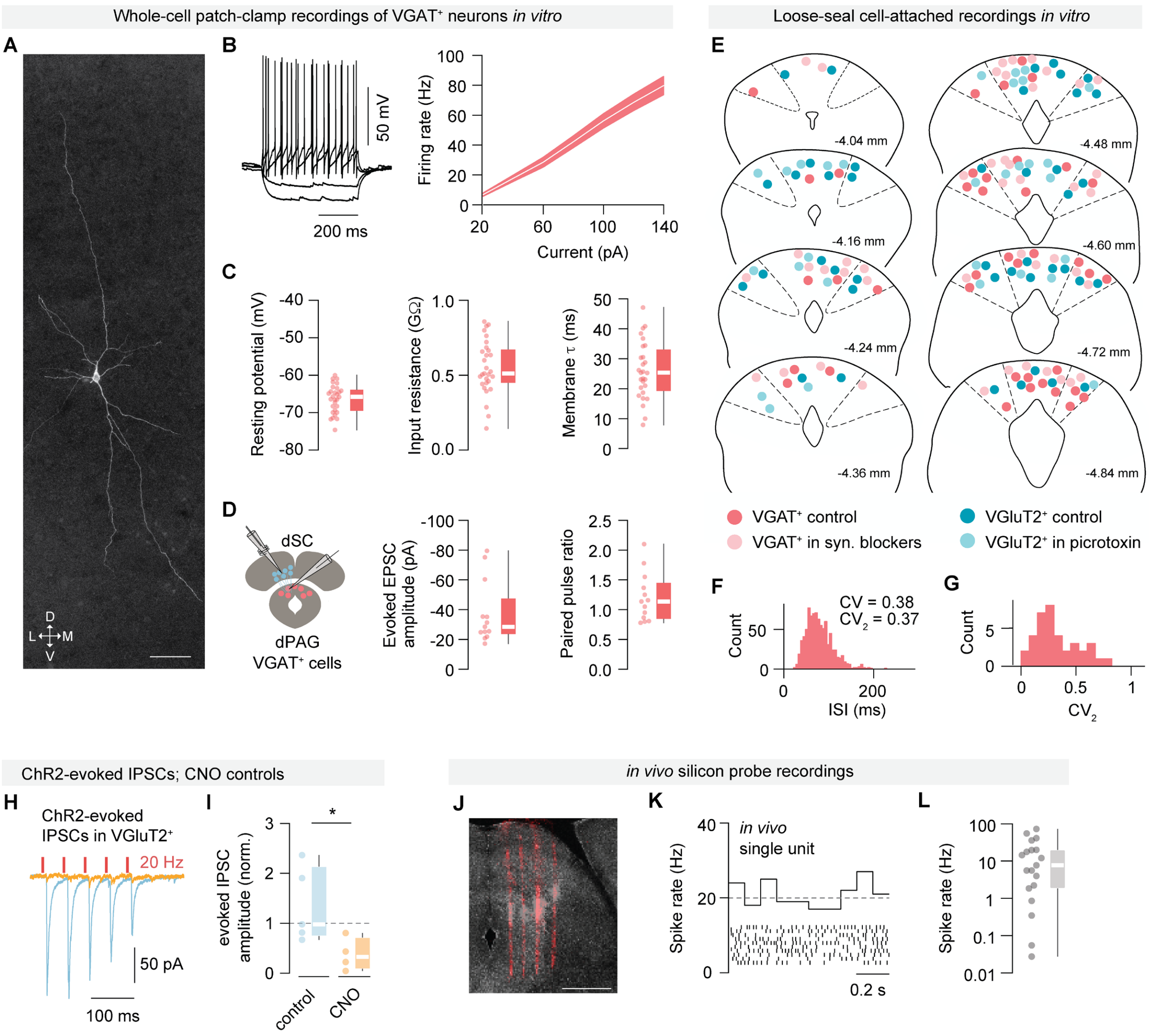

Figure S1. Electrophysiological properties of VGAT$^+$ dPAG neurons and IPSCs *in vitro,* and firing rates *in vivo.* Related to Figure 1.

A. Maximum intensity projection of a confocal stack of a biocytin-filled VGAT$^+$ neuron in the dPAG. Arrows indicate the neuron's orientation: D – dorsal, V – ventral, M – medial, L – lateral.

B. Left: membrane potential traces of a VGAT$^+$ dPAG neuron in response to current injections of different amplitudes. Right: summary plot of the current-firing rate relationship of VGAT$^+$ dPAG neurons (shaded area is s.e.m; n = 29 cells, N = 8 mice).

C. The mean resting membrane potential (-66.31 ± 0.69 mV), input resistance (0.55 ± 0.033 GΩ) and membrane time constant (25.61 ± 1.79 ms) were calculated for the same neurons as in B**.**

D. Electrical stimulation of the deeper layers of the superior colliculus (dSC; left panel: schematic of the experimental configuration) evokes excitatory postsynaptic currents in all tested VGAT$^+$ dPAG neurons with a mean peak amplitude of -37.07 ± 5.78 pA (peak amplitude of first pulse; middle panel) and a mean paired pulse ratio of 1.2 ± 0.11 (right panel) (n = 13 cells, N = 4 mice).

E. Loose-seal cell-attached recordings were made from identified VGAT$^+$ dPAG neurons (control, dark red circles: n = 39; in synaptic blockers, light red circles: n = 45) and VGluT2$^+$ neurons (control, dark blue circles: n = 42; in picrotoxin, light blue circles: n = 30). Superimposition of individual recorded

cells along the rostrocaudal axis of the PAG, coordinates are in mm and from bregma. Mouse brain images adapted from (Paxinos and Franklin, 2001).

F. Example ISI histogram of a regularly firing $VGAT^{+}$ neuron recorded *in vitro*.

G. Summary histogram of the mean CV_2 of all $VGAT^{+}$ dPAG cells recorded in control conditions.

H. Example trace of ChR2-evoked IPSCs recorded in a putative dPAG excitatory neuron (stimulation protocol: 1ms duration, 5 pulses, 20Hz) before (blue trace) and after application of 10 μM CNO (orange trace) to silence $VGAT^{+}$ dPAG neurons.

I. Summary plot of the normalized evoked IPSC amplitude in control conditions and after 10 μM CNO application (control: n = 5 cells; 10 μM CNO: n = 4 cells; N = 2 mice).

J. Example image of a coronal midbrain slice with the tracks of a 4-shank Neuropixels 2.0 probe made visible through DiI staining of the shanks prior to insertion into the brain.

K. Example firing of a single unit recorded in the dPAG *in vivo* during exploration with the mean spike rate (histogram) over time shown for 10 randomly sampled 1 s time intervals from within a 60 s time window. The grey dotted line indicates the mean spike rate (19.96 Hz) for the same neuron.

L. Average spike rate of all dPAG single units recorded *in vivo* (note log scale of y-axis). Each plotted data point (grey filled circles) is the mean firing rate of a single unit. Box-and-whisker plots show median, IQR and range, as well as individual data points.

Box-and-whisker plots show median, IQR and range, as well as individual data points.

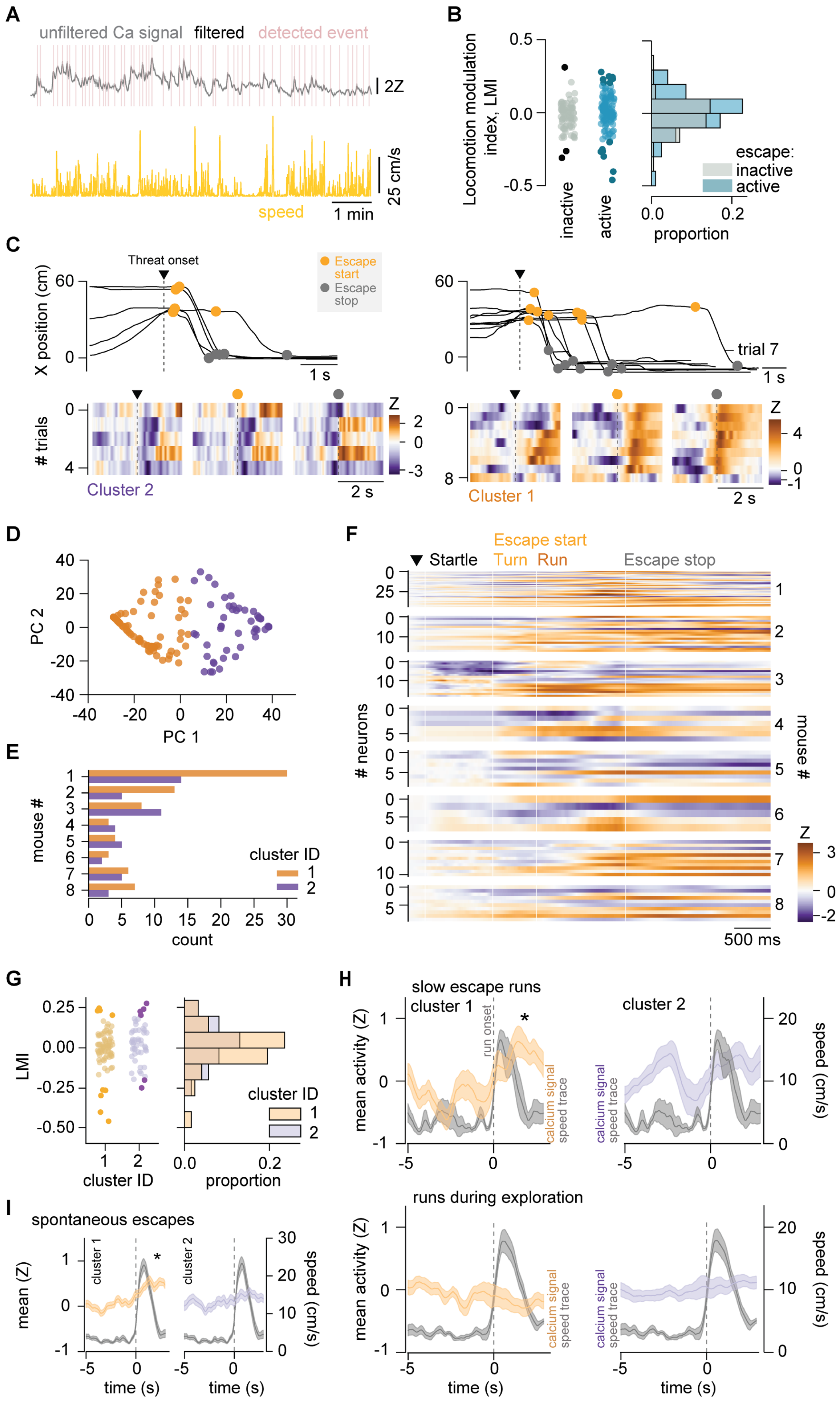

A
unfiltered Ca signal
filtered
detected event
2Z
speed
25 cm/s
1 min
B
Locomotion modulation index, LMI
0.5
0.0
-0.5
inactive
active
escape:
inactive
active
0.0
0.2
proportion
C
Threat onset
Escape start
Escape stop
X position (cm)
60
0
1 s
trial 7
trials
Z
2
0
-3
4
8
2 s
Cluster 2
Cluster 1
D
PC 2
PC 1
E
mouse #
count
cluster ID
F
Startle
Escape start
Turn
Run
Escape stop
neurons
mouse #
500 ms
G
LMI
cluster ID
proportion
H
slow escape runs
cluster 1
cluster 2
run onset
mean activity (Z)
speed (cm/s)
calcium signal
speed trace
runs during exploration
time (s)
I
spontaneous escapes
mean (Z)
time (s)

Figure S2. Analysis of calcium activity during exploration and spontaneous escapes and further examples of escape-active neurons. Related to Figure 2.

A. Single-neuron calcium signal (top) and animal's speed (bottom) over time, showing calcium events (pink lines) detected using the filtered signal (black line).

B. Distribution of cells' locomotion modulation index of activity during exploration for populations that are inactive and active at escape. Dark coloured dots show significantly modulated cells, determined by bootstrapping with resampling (95 % confidence interval significantly different from zero). Escape-active population contains a larger fraction of significantly modulated cells (8/123 and 7/123 cells, positive and negatively-modulated respectively) than the inactive population (1/76 and 2/76).

C. Each row shows (left panel) all escape trials of one session of one animal, showing animal's position along the long-axis of the rectangular arena (shelter entrance at X = 0 cm) over time, aligned to stimulus onset. Right: Heatmap of the calcium signal of one example neuron during the escape trials shown on the left, with the peak calcium signal aligned to threat-onset, escape onset (turn) and escape stop.

D. PC scores for mean time-warped signal during escape for each neuron, coloured by K-means cluster ID (n = 123 neurons, N = 8 animals).

E. Histogram of neurons assigned to clusters in D for each mouse.

F. Mean time-warped signal for each neuron (same data as in Figure 2E) for reach mouse.

G. Distribution of cells' locomotion modulation index of activity for the two escape-active clusters. Dark coloured dots denote significantly modulated cells, determined by bootstrapping with resampling (95 % confidence interval significantly different from zero). Both clusters contain similar fractions of positively and negatively modulated cells (cluster 1, 4/74 and 5/74 cells; cluster 2, (4/49 and 2/49), positive and negative modulation index respectively), and the means of the cluster distributions are not significantly different ($P = 0.08$, Mann-Whitney test).

H. Mean cluster population activity and animal speed trace for escape trials with slow peak run speed (top; mean peak speed 20.4 ± 1.9 cm/s) and fast running events (i.e., darting) during exploration by the same mice (bottom; mean peak speed, 20.2 ± 1.3 cm/s), aligned to the onset of running. Cluster 1 activity rises significantly during escape running but not exploratory running, while cluster 2 activity changes are not detected in either behaviour (*cluster 1, slow escapes*, mean signal -0.18 ± 0.20 Z and 0.39 ± 0.17 Z in the 2 s before and after run onset respectively, two-tailed paired t-test, $P = 0.039$, n = 18 neurons, 15 trials, N = 4 mice; *cluster 2, slow escapes*, mean signal -0.14 ± 0.21 Z before and 0.3 ± 0.16 Z after, two-tailed paired t-test, $P = 0.11$, n = 19 neurons, 15 trials, N = 4 mice; *cluster 1, runs during exploration*, mean signal -0.12 ± 0.12 Z before and -0.20 ± 0.13 Z after, two-tailed paired t-test, $P = 0.66$; n = 21 neurons, 8 trials, N = 4 mice; *cluster 2, runs during exploration,* mean signal -0.02 ± 0.11 Z before and 0.06 ± 0.11 Z after, two-tailed paired t-test, $P = 0.72$; n = 23 neurons, 8 trials, N = 4 mice).

I. Mean cluster population activity and animal speed trace aligned to the onset of spontaneous escapes to the shelter in the absence of sensory stimulation (cluster 1, mean signal 0.01 ± 0.07 Z and 0.42 ± 0.07 Z in the 2 s before and after escape onset respectively, two-tailed t-test $p < 0.0025$, n = 61 neurons, 14 trials, N = 7 mice; cluster 2, mean signal 0.10 ± 0.09 Z before and 0.24 ± 0.08 Z after, two-tailed t-test $p = 0.26$, n = 44 neurons, 13 trials, N = 7 mice).

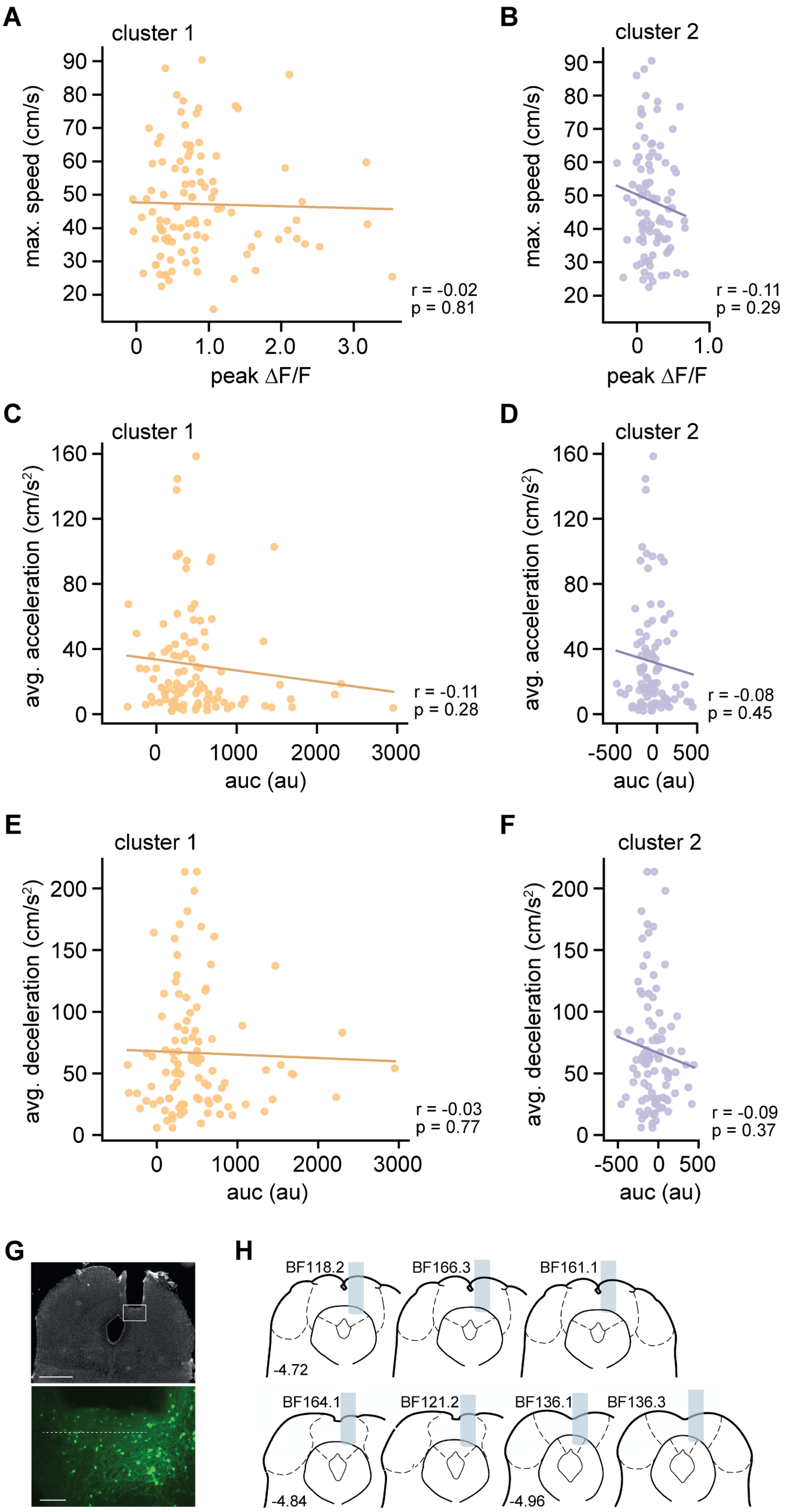
A
cluster 1
max. speed (cm/s)
peak ΔF/F
r = -0.02
p = 0.81
B
cluster 2
max. speed (cm/s)
peak ΔF/F
r = -0.11
p = 0.29
C
cluster 1
avg. acceleration (cm/s²)
auc (au)
r = -0.11
p = 0.28
D
cluster 2
avg. acceleration (cm/s²)
auc (au)
r = -0.08
p = 0.45
E
cluster 1
avg. deceleration (cm/s²)
auc (au)
r = -0.03
p = 0.77
F
cluster 2
avg. deceleration (cm/s²)
auc (au)
r = -0.09
p = 0.37
G
H
BF118.2
BF166.3
BF161.1
-4.72
BF164.1
BF121.2
BF136.1
BF136.3
-4.84
-4.96

Figure S3. Calcium activity during escape is not correlated with escape vigour, and gradient index lens placements in the dPAG for *in vivo* calcium imaging recordings. Related to Figure 2.

A. and **B.** Single trial mean peak Z-scored calcium activity during escape for cluster 1 neurons (A) and 2 (B) against maximum speed of the escape trial.

C. and **D.** Area of negative Z-scored calcium activity during escape for cluster 1 (C) and 2 (D), against peak escape acceleration from escape onset to time of maximum speed during escape.

E. and **F.** Absolute area of positive Z-scored calcium activity for cluster 1 (E) and 2 (F) against peak deceleration from time of maximum speed to escape termination. N = 100 trials, 8 animals; Spearman's r and p-values are indicated for each measure in the respective panel.

G. PFA-fixed, coronal section stained with DAPI showing a recovered GRIN lens track in the dPAG (top, scale bar = 500 µm) and a zoom-in on the imaged region of interest with GCaMP6s-expressing $VGAT^+$ neurons (bottom, scale bar = 100 µm, dashed line = approximate imaging plane).

H. Placements of recovered GRIN lenses along the rostrocaudal axis of the PAG (N = 7 out of 8 experiments). The images in A are from animal BF118.2.

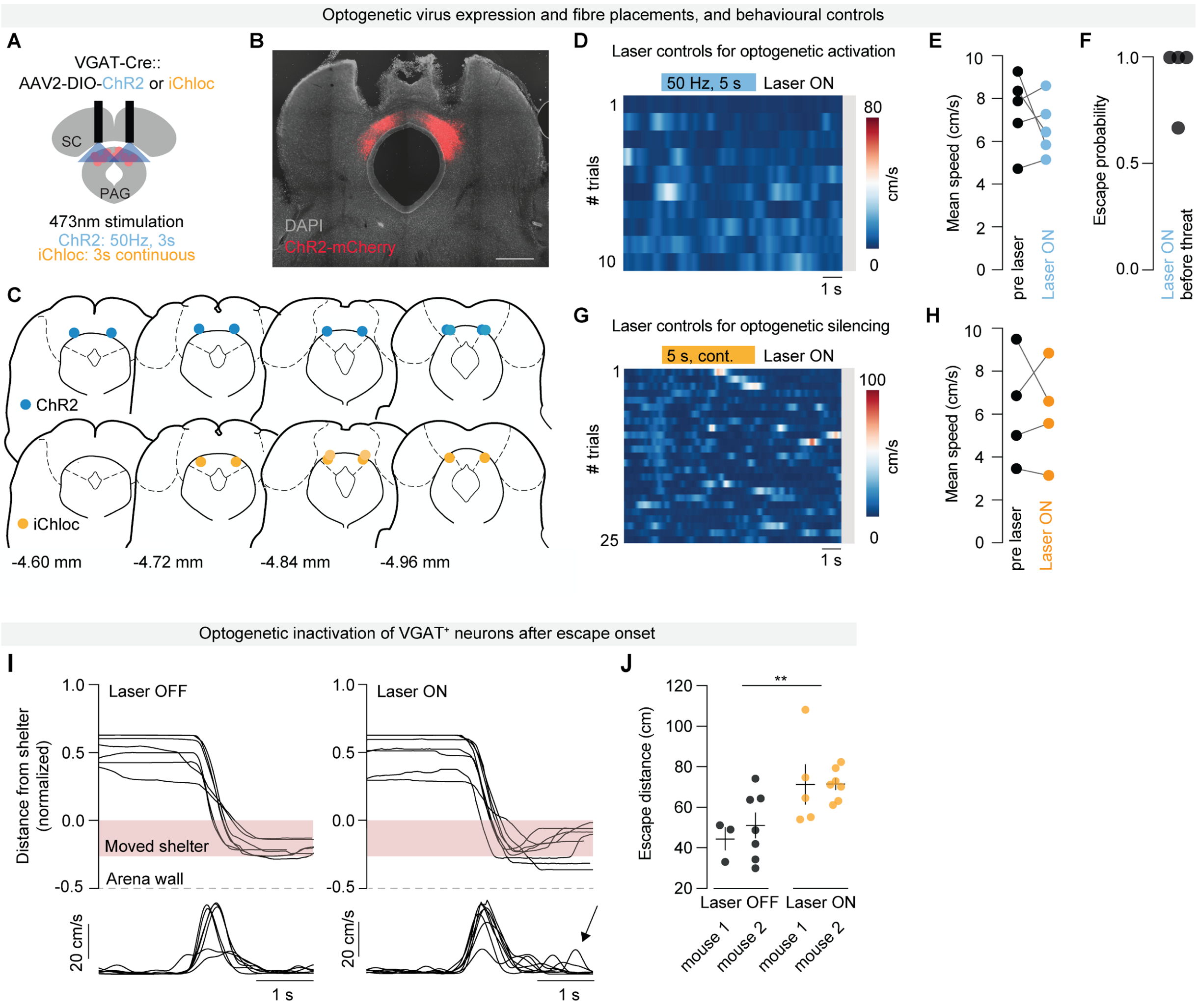

Figure S4. Optogenetic manipulation of VGAT^{+} dPAG neurons does not change movement speed during exploration, and optogenetic inhibition of VGAT^{+} neurons during escape increases escape distance. Related to Figure 3.

A. Schematic of the experimental design and optogenetic stimulation protocols (ChR2: 50 Hz, 3 s; iChloC: continuous, 3 s).

B. Coronal slice stained with DAPI (blue) with bilateral expression of AAV2/2-DIO-ChR2-mCherry (red) localised to the dPAG and optic fibre tracks. Scale bar = 1 mm.

C. Bilateral optic fibre placements along the rostrocaudal axis of the dPAG are shown for ChR2 (blue, top; N = 5 mice) and iChloC experiments (orange, bottom; N = 4 mice). Rostrocaudal position from Bregma, based on Paxinos and Franklin.

D. Example speed raster plot during optogenetic stimulation of VGAT^{+} dPAG neurons during exploratory movement. Grey bar on the right side of the histogram indicates no escapes. Laser duration is shown in blue.

E. Summary plot of mean speed before and during laser stimulation (ChR2; N = 5 mice, n = 92 trials).

F. Animals escape to threat stimuli when optogenetic stimulation of VGAT^{+} dPAG neurons precedes and ends immediately before threat stimulation (N = 4 mice, P = 0.44, Mann-Whitney test between probability of escape when laser precedes vs no laser condition).

G. The same as D, for optogenetic inhibition. Laser duration shown in orange.

H. Mean speed before and during laser stimulation for optogenetic inhibition (iChloC; N = 4 mice, n = 108 trials).

I. Left: moving the shelter location in the arena away from the arena wall leads to sensory-evoked escapes to the new shelter location (indicated in light red). Control trials (Laser OFF) from one mouse during escape to shelter with normalized tracking coordinates over time (top panel) and the corresponding speed traces (bottom panel). Right: same as left panel, for Laser ON trials with light-activation of iChloC after escape onset. The same animal displays continued movement after reaching the shelter (black arrow).

J. Summary plot of the total escape distance for Laser OFF trials (10 trials) and Laser ON trials (13 trials) showing a significant increase during light-activation of iChloC (N = 2 mice). Plot is showing the mean ± s.e.m and individual trials, separated by mouse.
